# Oral octanoylcarnitine alleviates exercise intolerance in mouse models of long-chain fatty acid oxidation disorders

**DOI:** 10.1172/jci.insight.199443

**Published:** 2025-10-16

**Authors:** Keaton J. Solo, Yuxun Zhang, Sivakama S. Bharathi, Bob B. Zhang, Adam C. Richert, Alexandra V. Schmidt, Clinton Van’t Land, Olivia D’Annibale, Timothy C. Wood, Eric S. Goetzman

**Affiliations:** 1 Department of Pediatrics, Division of Genetic and Genomic Medicine, University of Pittsburgh School of Medicine, Pittsburgh, Pennsylvania, USA.; 2 Department of Pediatrics, University of Colorado Anschutz Medical Campus/Children’s Hospital of Colorado, Aurora, Colorado, USA.

**Keywords:** Genetics, Metabolism, Bioenergetics, Monogenic diseases, Therapeutics

## Abstract

Long-chain fatty acid oxidation disorders (LC-FAODs) cause energy deficits in heart and skeletal muscle that are only partially corrected by current medium-chain lipid therapies such as triheptanoin. We find that heart and muscle lack medium-chain acyl-CoA synthetases, limiting the capacity for β-oxidation of medium-chain fatty acids. Instead, heart and muscle mitochondria robustly respire on medium-chain acylcarnitines. The mitochondrial matrix enzyme carnitine acetyltransferase (CrAT) efficiently converts orally delivered octanoylcarnitine (C_8_-carnitine) to octanoyl-CoA for energy generation. C_8_-carnitine exhibits twice the oral bioavailability of triheptanoin and distributes to muscle and heart. A single oral dose significantly enhances grip strength and treadmill endurance while attenuating lactic acidosis in 2 mouse models of LC-FAODs. Thus, medium-chain acylcarnitines overcome a previously unrecognized metabolic bottleneck in LC-FAOD muscle and may represent an alternative to triglyceride-based therapies for bioenergetic disorders.

## Abstract 2

Oral C_8_-carnitine bypasses a muscle energy bottleneck, fuels mitochondria via CrAT, and improves strength and endurance in mouse models of fatty acid oxidation disorders.

## Graphical abstract

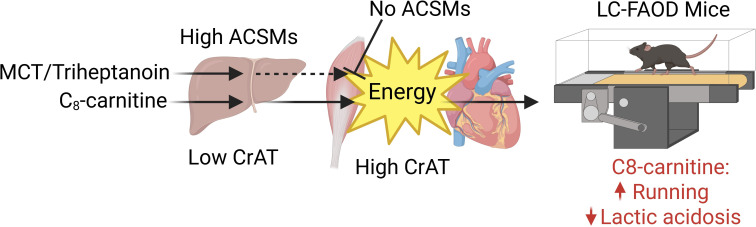

## Introduction

Long-chain fatty acid oxidation disorders (LC-FAODs) are a group of rare, inherited metabolic conditions caused by defects in mitochondrial enzymes required for the β-oxidation of long-chain fatty acids. There are 2 subgroups of LC-FAODs. The first group includes defects in the carnitine shuttle, which facilitates transport of long-chain fatty acids into the mitochondria. This group includes mutations in the genes carnitine palmitoyltransferase-1a (CPT1a), carnitine-acylcarnitine translocase (CACT), and carnitine palmitoyltransferase-2 (CPT2). The second group includes mutations in mitochondrial matrix β-oxidation enzymes, most notably very long-chain acyl-CoA dehydrogenase (VLCAD) and mitochondrial trifunctional protein (TFP). The 2 LC-FAOD subgroups exhibit largely overlapping symptoms due to impaired energy production, particularly during periods of fasting or increased energy demand (1, 2). The major organs affected in patients during infancy are liver and heart. Depending on the severity of the mutation, mortality during this period can be high. Later, in adolescence, muscle dysfunction becomes the dominant symptom moving into adulthood. Most adult patients suffer recurring, life-threatening bouts of rhabdomyolysis, increasing the disease burden on both patients and health care systems (1, 3).

Current treatment for LC-FAODs consists of frequent feeding and replacement of dietary long-chain fats with medium-chain fats (4). For more than 40 years, medium-chain fats were provided as medium-chain triglycerides (MCT) containing C_8_ fatty acids. The current understanding of MCT oil, which currently has a ~$2.5 billion annual market as a supplement for weight management and sports nutrition, is that the C_8_ fatty acids traverse the mitochondrial membrane without the need for facilitated transport by the carnitine shuttle and rapidly produce energy via β-oxidation (5). In LC-FAODs, MCT adequately manages liver symptoms but often fails to address cardiomyopathy and rhabdomyolysis (6). This was believed to be caused by chronic depletion of TCA cycle intermediates (7). The proposed solution was to replace the C_8_ fatty acids with C_7_ fatty acids, which, after 2 rounds of chain-shortening, produce a C_3_ remnant which is converted to succinate, thereby refilling the TCA cycle (anaplerosis) (7). Consequently, the preferential treatment for LC-FAODs is now triheptanoin, a C_7_ triglyceride. Triheptanoin is believed to possess the rapid energy production properties of MCT with the additional benefit of anaplerosis (8). Clinical trials and case studies demonstrate that triheptanoin improves symptoms for many patients with LC-FAOD (9, 10). However, there are limitations to the utility of triheptanoin. First, it is not efficacious against the cardiomyopathy seen in patients with severe, neonatal onset LC-FAODs, a subgroup that continues to exhibit high mortality (11). Second, triheptanoin is only partially effective against the rhabdomyolysis that dominates the clinical picture for patients who live past childhood (10). And third, the rate of treatment-related adverse events with triheptanoin is very high, at ~85%. Diarrhea, abdominal pain, and vomiting caused by triheptanoin oil reduce the quality of life for many patients (10).

New therapies are needed that focus on reducing the symptoms of rhabdomyolysis in LC-FAODs. In the present studies, we used LC-FAOD mouse models to interrogate the metabolism of MCT and triheptanoin in muscle and heart. Our work identifies 2 major barriers to the use of MCT or triheptanoin as a therapy for LC-FAOD muscle symptoms. First, the bulk of medium-chain lipids taken orally are consumed by the liver. Second, and most importantly, muscle and heart have a low capacity for activating medium-chain fatty acids to coenzyme A (CoA), which is a prerequisite step for fatty acid β-oxidation. In short, our data challenge the current understanding of MCT and triheptanoin metabolism for organs outside the liver. We further show that there is an alternative route to convert medium-chain fatty acids to their acyl-CoA forms in muscle, which is by supplying them as acylcarnitine esters and leveraging carnitine acyltransferase enzymes to exchange the carnitine for CoA. Finally, we establish the efficacy of acylcarnitine therapy for treating exercise intolerance in mouse models of LC-FAODs.

## Results

### MCT fail to improve exercise capacity in an LC-FAOD mouse model.

**Figure 1:**
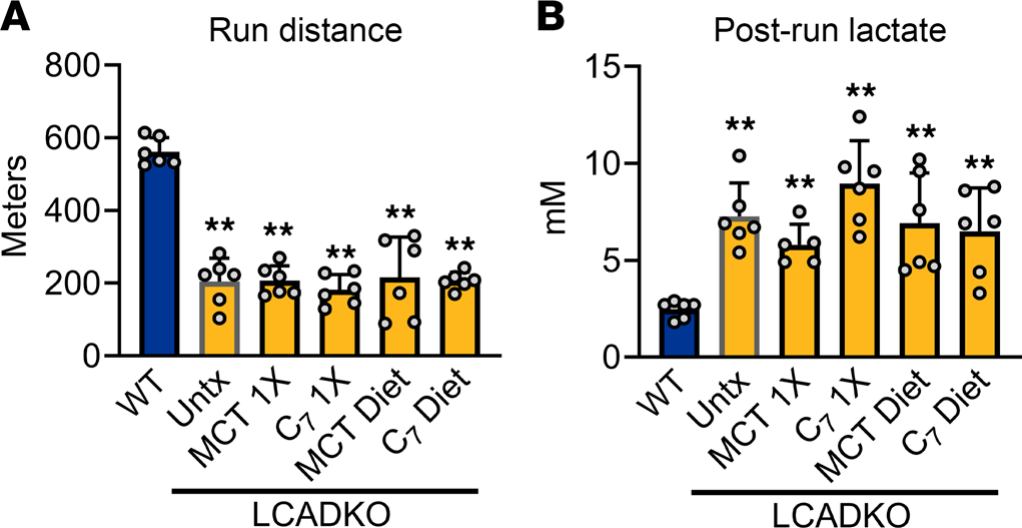
Medium-chain triglycerides fail to improve exercise capacity in an LC-FAOD mouse model. Male LCAD-KO age 12–16 weeks, and matched WT controls, were run to exhaustion on a treadmill. (**A** and **B**) Running distance (**A**) and postrun blood lactate (**B**) were recorded. Cohorts of LCAD-KO mice were either untreated (Untx) or treated with medium-chain triglycerides (MCT) or triheptanoin (C_7_). MCT and C_7_ were given either as 0.5 mg/g oral boluses (1×) or delivered as 35% of calories in pelleted diets for 10 days. None of the treatments improved running distance or lactic acidosis. **P < 0.01 for all LCAD-KO groups versus WT controls. Data are shown as mean ± SEM.

In mice, long-chain acyl-CoA dehydrogenase (LCAD) is the dominant acyl-CoA dehydrogenase family member, fulfilling the role that VLCAD plays in humans (12–14). Correspondingly, LCAD-KO mice model the symptoms of human VLCAD deficiency, including hypoketotic hypoglycemia and cardiomyopathy (14, 15). Here, we established that LCAD-KO mice also exhibit multiple signs of muscle dysfunction, such as significantly reduced basal locomotion, reduced grip strength, and reduced latency to fall on the hanging wire test (Supplemental Figure 1; supplemental material available online with this article; https://doi.org/10.1172/jci.insight.199443DS1). When exercise-naive LCAD-KO mice are challenged with a run-to-exhaustion protocol, they run only one-third the distance of their WT counterparts (Figure 1A). Despite running a much shorter distance than WT mice, LCAD-KO mice display postrun lactic acidosis (Figure 1B). Prerun blood glucose, prerun blood lactate, and postrun blood glucose values are not different from WT (Supplemental Figure 2). These data suggest an increased reliance upon glycolysis for energy during exercise. We then tested whether medium-chain lipid therapies could improve exercise performance in the LCAD-KO mouse model. Patients with LC-FAOD consume MCT oil or triheptanoin 3–4 times per day at ~0.5 g/kg, with an overall target of ~35% of total calories (10). To mimic this dosing, LCAD-KO mice were gavaged with 0.5 mg/g of MCT oil or triheptanoin 20 minutes prior to a treadmill challenge. Neither compound improved running distance or postrun lactic acidosis (Figure 1, A and B). We also tested the effect of chronic feeding by incorporating MCT and triheptanoin into pelleted mouse diets at 35% of total calories. LCAD-KO mice were fed these diets for 10 days and then challenged with acute exercise. Again, neither compound improved running distance nor ameliorated the postrun lactic acidosis (Figure 1, A and B).

### Exogenous medium-chain fatty acids are poorly metabolized outside of liver.

**Figure 2:**
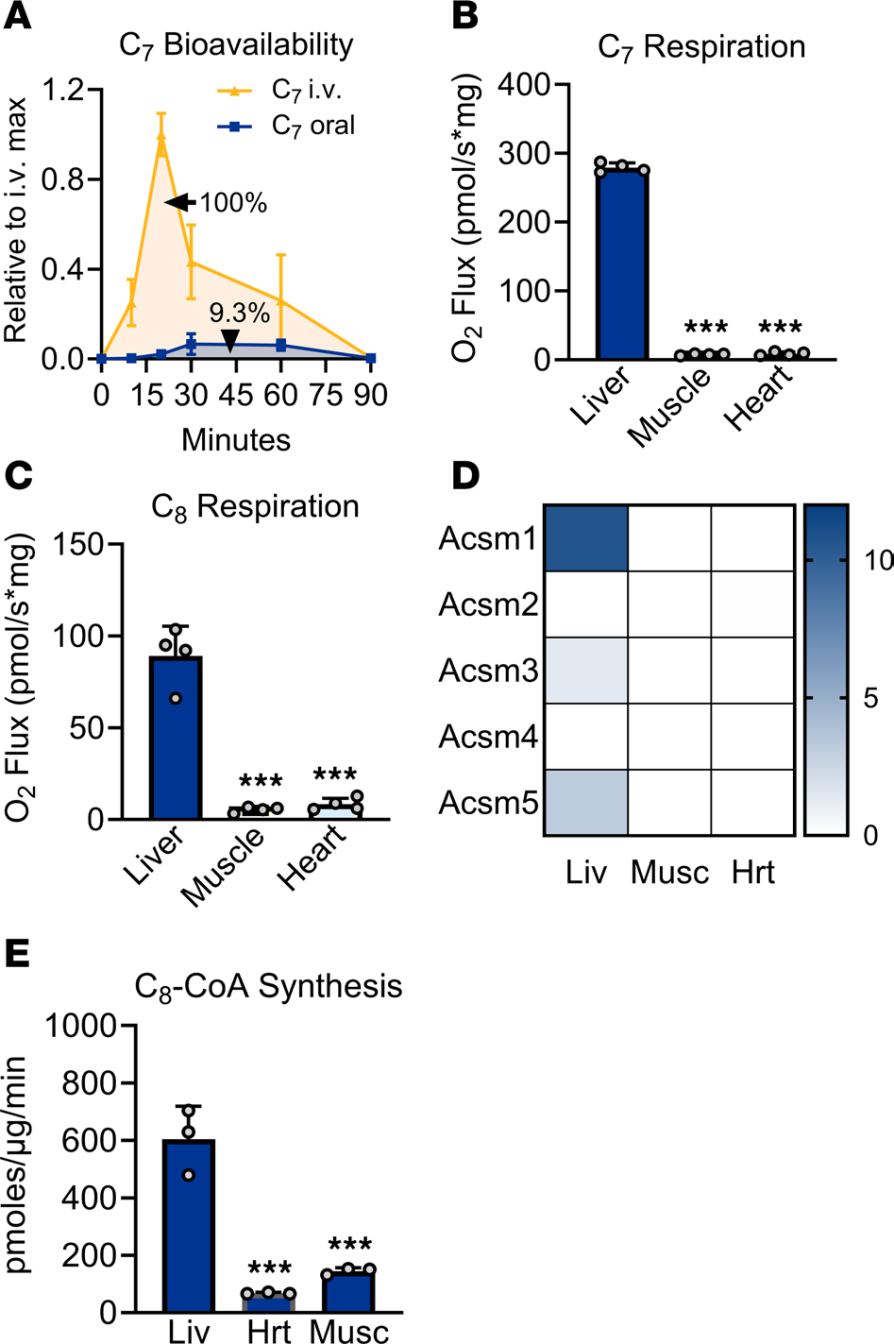
Exogenous medium-chain fatty acids are poorly metabolized outside of liver. (**A**) Oral bioavailability of triheptanoin was determined by dosing *n* = 3 male rats with 0.5 mg/g and following C_7_ fatty acid levels in the blood over time. The ratio of the area under the curve for oral dose: i.v. dose represents bioavailability, which is 9.3%. (**B** and **C**) Mitochondria were isolated from the indicated WT mouse tissues and used for Oroboros respirometry studies to determine oxygen consumption on medium-chain free fatty acids C_7_ and C_8_. (**D**) A previously published proteomics dataset (20) was interrogated for expression levels of the 5 medium-chain acyl-CoA synthetase (ACSM) isoforms. (**E**) ^14^C-labeled C_8_ was followed to ^14^C_8_-CoA in the presence of mitochondria isolated from WT mouse liver, heart (Hrt), or muscle (Musc). ***P < 0.001, heart and muscle versus liver. Data are shown as mean ± SEM.

We set out to determine why medium-chain therapies were ineffective for treating exercise intolerance in the LCAD-KO mouse. First, we interrogated absorption and first-pass liver metabolism. It is generally held that oral medium-chain lipids are trafficked from the gut into portal circulation (16), but empirical data on the degree of first-pass liver metabolism of these lipids has been conflicting (17–19). Furthermore, little is known about the absorption of triheptanoin, which has supplanted MCT as the therapy of choice for LC-FAODs. We interrogated the oral bioavailability of triheptanoin using rats with an indwelling jugular port. Rats were orally gavaged with triheptanoin or infused with the same amount of C_7_ fatty acids via the jugular port. Blood was sampled from the port at time points from 0 to 90 minutes. Blood lipids were hydrolyzed to convert any absorbed triheptanoin into free C_7_, which was then quantified by mass spectrometry. Oral bioavailability for triheptanoin was calculated as the ratio of area under the curve (AUC) for oral dosing to the AUC for i.v. dosing. Triheptanoin was observed to be ~9% orally bioavailable (Figure 2A), meaning 9% of the C_7_ reaches systemic circulation. Thus, low bioavailability likely contributed to the lack of therapeutic effect observed in the LCAD-KO mouse model.

We next asked, in the event systemic distribution could be improved, which medium-chain fatty acid would be preferred by the target organs muscle and heart. Oroboros mitochondrial respirometry was used to evaluate utilization of C_7_ and C_8_ by WT mouse muscle and heart mitochondria, with liver mitochondria serving as a benchmark control. Notably, both muscle and heart mitochondria exhibited very low capacities for oxidizing free C_7_ or free C_8_ fatty acids, as compared with liver (Figure 2, B and C). To determine the mechanism behind this striking discrepancy, we analyzed our recently published WT mouse proteomics dataset for expression of the medium-chain FAO machinery in liver, muscle, and heart (20). All 3 tissues express the required enzymes for medium-chain FAO including medium-chain acyl-CoA dehydrogenase (MCAD), enoyl-CoA hydratase (ECHS1), hydroxyacyl-CoA dehydrogenase (HAD), and the medium-chain ketoacyl-CoA thiolase (THIM) (Supplemental Figure 3). However, muscle and heart completely lacked expression of the medium-chain acyl-CoA synthetases (ACSMs), which are required to activate medium-chain fatty acids to CoA for FAO (Figure 2D). There are 5 ACSM isoforms. ACSM2 is kidney specific, and ACSM4 is gut specific (21–23). Liver expresses the remaining 3 ACSMs (ACSM1, -3, and -5), but none of these were detected in muscle or heart. Immunoblotting corroborated these results for ACSM1 and ACSM3 (Supplemental Figure 4). Muscle, but not heart, exhibited potential low-level expression of ACSM5 (Supplemental Figure 4). In an ACSM activity assay, based on conversion of ^14^C-C_8_ into ^14^C-C_8_-CoA, WT muscle and heart homogenates exhibited 10%–20% capacity for C_8_-CoA synthesis compared with liver homogenates (Figure 2E).

### Heart and muscle mitochondria prefer medium-chain acylcarnitines over medium-chain fatty acids.

**Figure 3:**
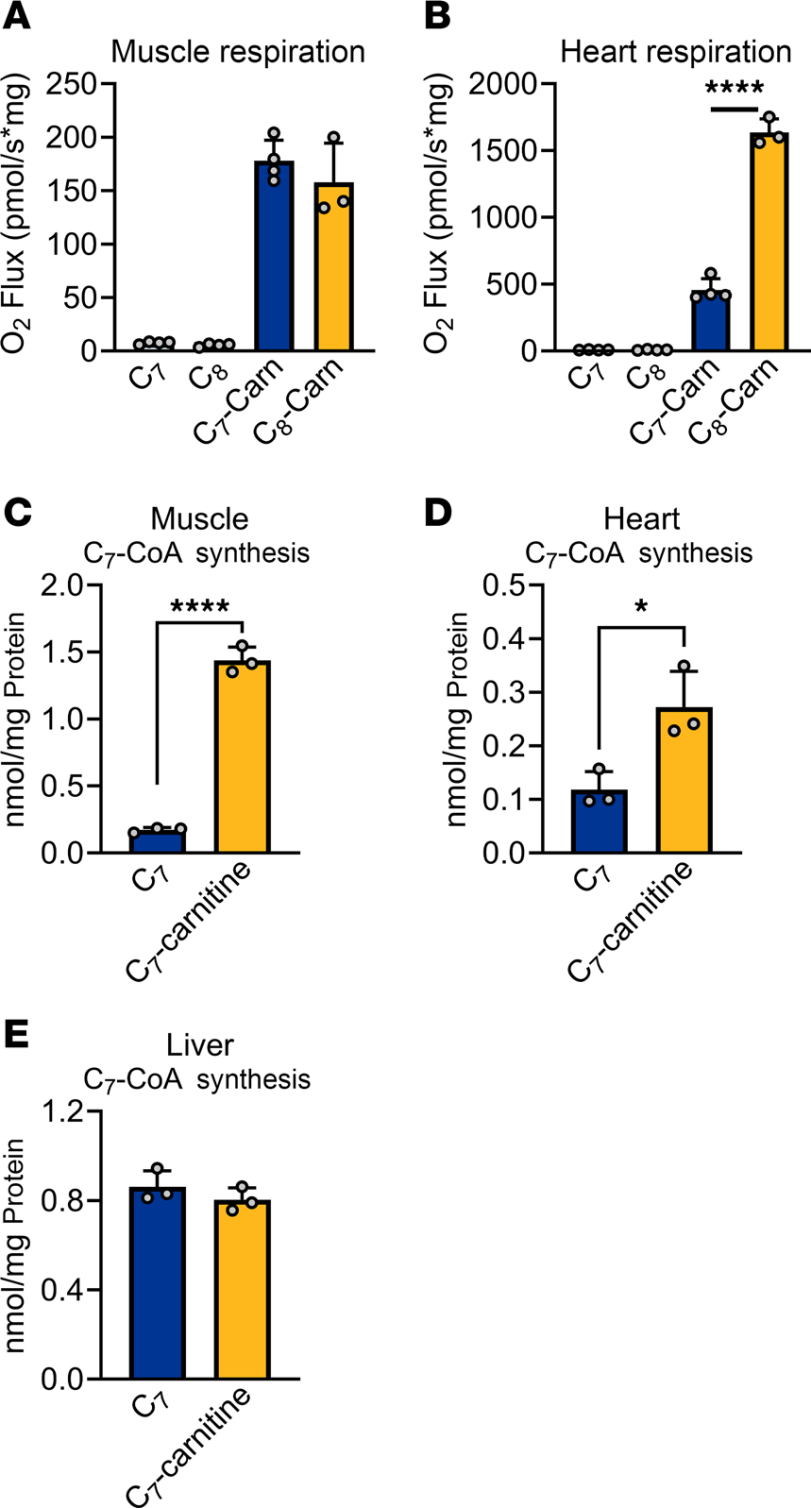
Heart and muscle mitochondria prefer medium-chain acylcarnitines over medium-chain fatty acids. (**A** and **B**) WT muscle and heart mitochondria were isolated and probed for respiratory capacity in the presence of either medium-chain free-fatty acids (C_7_, C_8_) or their acylcarnitine conjugates (C_7_-Carn, C_8_-Carn) using the Oroboros Oxygraph-2K. (**C**–**E**) WT mouse tissue homogenates were incubated with C_7_ or C_7_-carnitine. Mass spectrometry was used to quantify the amount of C_7_-CoA formed in 1 hour. *P < 0.05; ****P < 0.0001 by Student’s *t* test. Data are shown as mean ± SEM.

Pereyra et al. (24) recently demonstrated that muscle and heart mitochondria have the capacity to oxidize medium-chain FA, but they must do so via the carnitine shuttle, like long-chain fatty acids. This is in keeping with our observation that muscle and heart lack ACSM expression. ACSMs are mitochondrial matrix enzymes. In liver, where ACSMs are abundantly expressed, medium-chain FA can flip-flop across the mitochondrial membranes and be activated to acyl-CoA in the matrix for FAO. In muscle and heart, the only path to FAO would be via long-chain ACSLs, which are known to have weak activity with medium-chain substrates (25, 26). ACSLs are all extramitochondrial, or on the outside of the mitochondria facing the cytosol, and any medium-chain acyl-CoA formed by these enzymes would be forced to access the mitochondrial matrix through the carnitine shuttle. We therefore reasoned that muscle and heart mitochondria would be capable of oxidizing medium-chain acylcarnitines for energy. Indeed, both muscle and heart mitochondria robustly respired on C_7_-carnitine and C_8_-carnitine, although heart had a clear preference for C_8_-carnitine (Figure 3, A and B). Such respiration would require the action of a carnitine acyltransferase enzyme, such as CPT2, to convert the metabolically inert medium-chain acylcarnitines into acyl-CoAs for oxidation. WT mouse heart and muscle homogenates convert C_7_-carnitine to C_7_-CoA at a much faster rate than they convert free C_7_ to C_7_-CoA (Figure 3, C and D). In contrast, liver shows no preference and equally converts C_7_ and C_7_-carnitine into C_7_-CoA (Figure 3E).

### Muscle and heart mitochondria use CrAT to metabolize medium-chain acylcarnitines.

**Figure 4:**
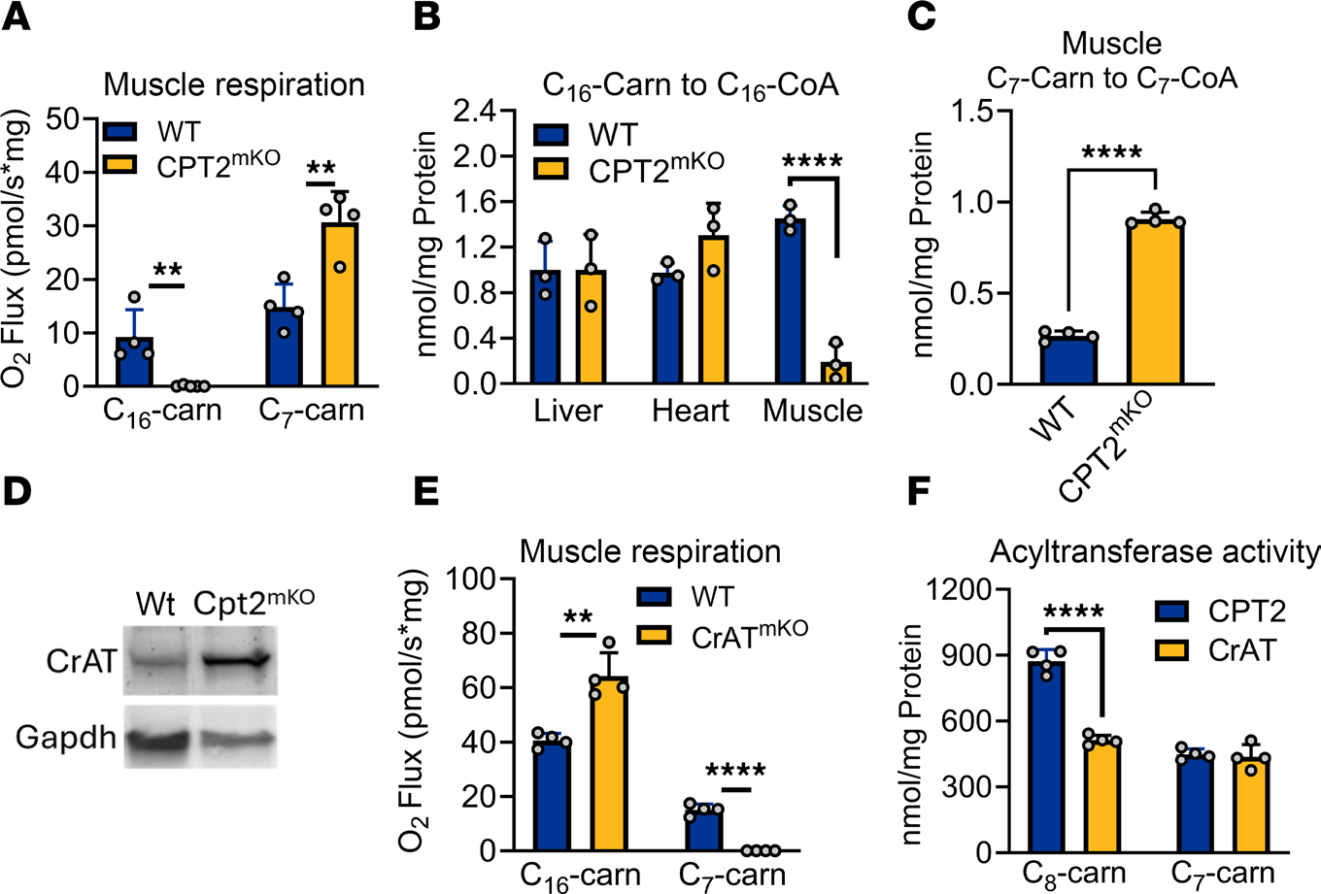
Muscle and heart mitochondria use CrAT to metabolize medium-chain acylcarnitines. (**A**) Soleus muscle mitochondria isolated from CPT2 muscle-specific KO mice (CPT2^mKO^) cannot respire on C_16_-carnitine, the preferred substrate for CPT2, but respire robustly on C_7_-carnitine. (**B**) The tissue specificity of the CPT2^mKO^ allele is demonstrated by the ability of liver and heart to normally convert C_16_-carnitine to C_16_-CoA, while muscle has near-zero capacity for this conversion. (**C**) CPT2^mKO^ soleus muscle mitochondria, while unable to convert C_16_-carnitine to C_16_-CoA (**B**), robustly convert C_7_-carnitine to C_7_-CoA. (**D**) The significant increase in acyl-CoA generating capacity in **C** is likely due to a compensatory increase in soleus muscle CrAT in the CPT2^mKO^ mouse. (**E**) Soleus muscle mitochondria isolated from CrAT muscle-specific KO mice (CrAT^mKO^) show the opposite result from CPT2^mKO^ muscle — i.e., high respiration on C_16_-carnitine but near-zero respiration on C_7_-carnitine. (**F**) The intact mitochondria experiments shown in **A** and **E** indicate that CrAT is the major contributor to C_7_-carnitine conversion to C_7_-CoA, but in vitro*,* both CPT2 and CrAT are active with medium-chain acylcarnitine substrates. **P < 0.01; ****P < 0.0001 by Student’s *t* test. Data are shown as mean ± SEM.

The carnitine shuttle canonically consists of CPT1, CACT, and CPT2 (2). CPT1 converts acyl-CoAs to acylcarnitines at the outer membrane. The acylcarnitines are imported through the inner membrane by CACT, and then CPT2 reconverts them to acyl-CoAs for oxidation. We anticipated that CPT2 would be required for oxidation of medium-chain acylcarnitines by muscle and heart mitochondria. Unexpectedly, muscle from CPT2 muscle-specific conditional KO mice (CPT2^mKO^) exhibited higher, not lower, C_7_-carnitine oxidation than floxed littermate controls (Figure 4A). The functional efficiency of the KO was confirmed by a near-complete loss of mitochondrial respiration on the long-chain substrate palmitoylcarnitine (C_16_-carnitine). Furthermore, in the carnitine acyltransferase enzyme activity assay, muscle from CPT2^mKO^ mice showed a severely limited capacity to convert C_16_-carnitine to C_16_-CoA, while liver and heart from the same animals had a normal capacity for this reaction (Figure 4B). In agreement with the Oroboros respirometry data, muscle homogenates from CPT2^mKO^ mice converted C_7_-carnitine to C_7_-CoA at an accelerated rate compared with WT muscle (Figure 4C). Together, these data indicate that CPT2 is not required for mitochondria to oxidize exogenous medium-chain acylcarnitines. Heart and muscle are known to highly express a related enzyme, carnitine acetyltransferase (CrAT), which interconverts acetyl-CoA and acetylcarnitine (27). Perhaps as a compensatory mechanism, CrAT expression is increased in CPT2^mKO^ muscle (Figure 4D). To test whether CrAT is responsible for mediating the oxidation of medium-chain acylcarnitines, we generated muscle-specific CrAT KO mice (CrAT^mKO^). CrAT^mKO^ muscle mitochondria respired robustly on the CPT2 substrate C_16_-carnitine but could not respire on C_7_-carnitine (Figure 4E). CrAT’s optimal activity is with C_2_ substrates, but some evidence exists for activity with longer-chain substrates up to C_8_ (28, 29). Using recombinant CrAT and CPT2, we confirmed that both enzymes can convert C_7_ and C_8_ acylcarnitines into their acyl-CoA counterparts in vitro (Figure 4F). The inability of CPT2 to facilitate medium-chain acylcarnitine oxidation in CrAT^mKO^ mouse muscle in vivo, despite in vitro activity with medium-chain acylcarnitines, suggests a potential compartmentalization of medium-chain and long-chain acylcarnitine metabolism in intact mitochondria.

### Oral C_8_-carnitine is rapidly absorbed, distributes to target organs, and improves exercise capacity in LCAD-KO mice.

**Figure 5:**
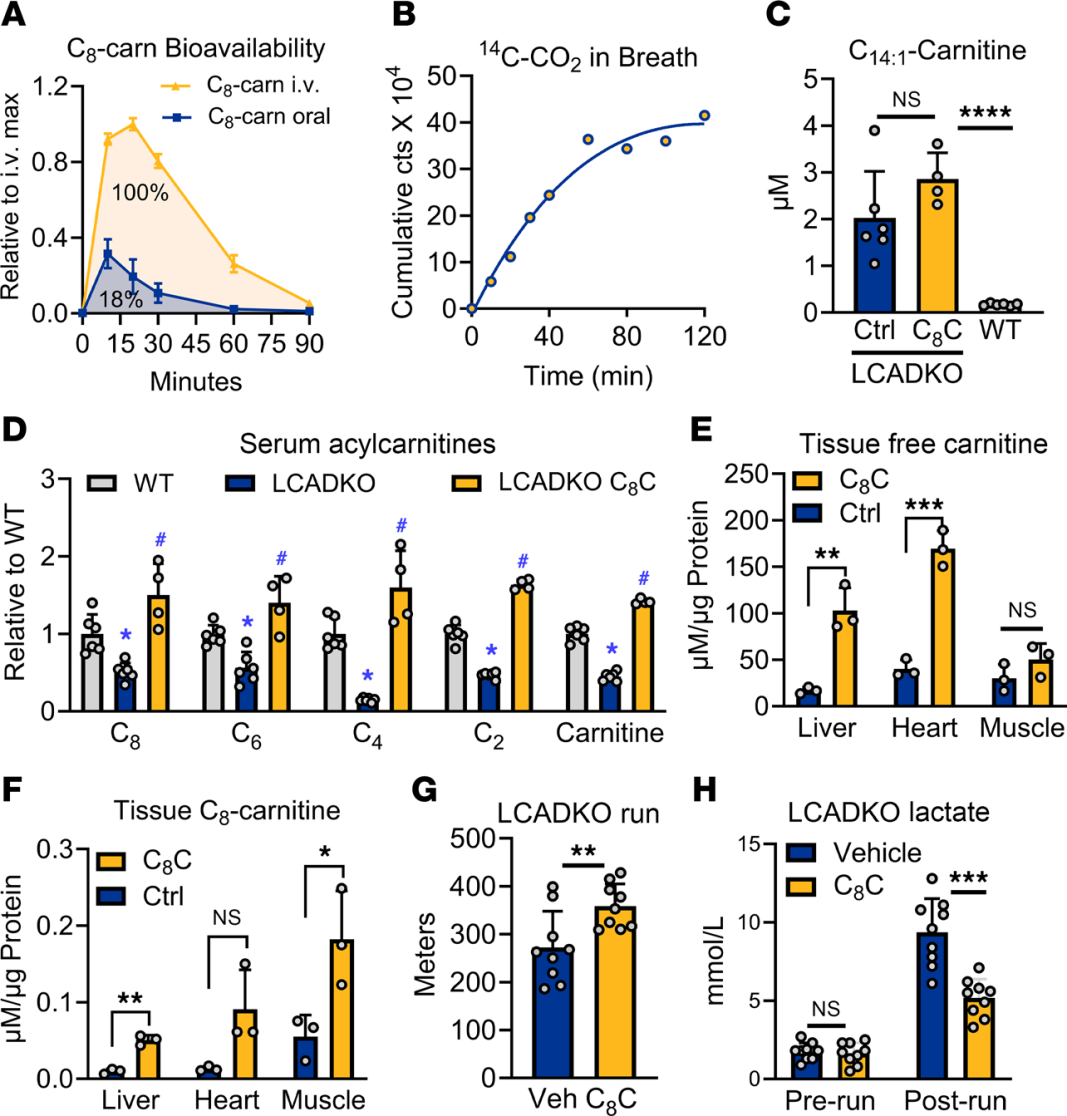
Oral C_8_-carnitine is rapidly absorbed, distributes to target organs, and improves exercise capacity in LCAD-KO mice. (**A**) Oral bioavailability of C_8_-carnitine was determined by dosing *n* = 3 male rats with 0.5 mg/g and following C_8_-carnitine levels in the blood over time. The ratio of the AUC for oral dose: i.v. dose represents bioavailability, which is 18%. (**B**) Data are averages of 2 male WT mice gavaged with 3 μCi of ^14^C-labeled C_8_-carnitine. Mice were placed into boxes connected to a KOH trap to capture exhaled breath. Samples withdrawn from the trap at the indicated times were subjected to scintillation counting. The *y* axis is the cumulative counts over time. When the curve plateaus, it means the substrate has been completely oxidized. (**C**–**F**) Effect of feeding control diet (Ctrl) versus C_8_-carnitine (C_8_C) at 3% w/w for 10 days on serum and tissue acylcarnitines. (**C**) Serum C_14:1_-carnitine is pathognomonic for human VLCAD deficiency and is recapitulated in the LCAD-KO mouse. Dietary C_8_C does not alter this diagnostic long-chain acylcarnitine species. (**D**) Serum acylcarnitines < 8 carbons are systemically reduced in LCAD-KO mice (blue vs. gray bars); dietary C_8_C increases all to normal or above normal levels (yellow bars). (**E** and **F**) Tissue levels of free carnitine and C_8_-carnitine are increased in the disease-relevant tissues liver, muscle, and heart after feeding C_8_-carnitine. (**G** and **H**) Single gavaged doses of 0.5 mg/g C_8_-carnitine improve running capacity in LCAD-KO mice while limiting lactic acidosis. (**D**) *P < 0.01 LCAD-KO versus WT and ^#^*P* < 0.01 LCAD-KO C_8_C versus WT; all other panels,*P < 0.05; **P < 0.01; ***P < 0.001; ****P < 0.0001 by Student’s *t* test with Bonferroni correction for multiple comparisons. Data are shown as mean ± SEM.

The studies above suggested the possibility that medium-chain acylcarnitines may have therapeutic value as an alternative energy source for LC-FAODs. We therefore followed the fate of orally administered C_8_-carnitine. Using rats with jugular ports, as was done with triheptanoin in Figure 2A, we established that 18% of orally dosed C_8_-carnitine reaches systemic circulation intact (Figure 5A). Thus, C_8_-carnitine is about 2× more orally bioavailable than triheptanoin. Blood levels peaked at 10 minutes after dosing, and the C_8_-carnitine was rapidly cleared. Similarly, when ^14^C-C_8_-carnitine tracer was administered orally, ^14^C-CO_2_ appeared in the exhaled breath within 10 minutes and metabolism was virtually complete after 1 hour (Figure 5B).

Next, LCAD-KO mice were fed with either standard diet (Ctrl) or standard diet supplemented with 3% w/w C_8_-carnitine for 10 days. Serum, muscle, heart, and liver tissue were subjected to acylcarnitine profiling. Patients with LC-FAOD accumulate long-chain acylcarnitines in the blood, which is the basis of newborn screening programs for these disorders (30). In particular, increased serum C_14:1_-carnitine is pathognomonic for VLCAD deficiency and is also seen in LCAD-KO mice as a model of VLCAD deficiency (14, 15). As expected, 10 days of dietary C_8_-carnitine did not significantly alter serum C_14:1_-carnitine levels in LCAD-KO mice (Figure 5C). However, dietary C_8_-carnitine increased the levels of downstream short-chain acylcarnitines, including free carnitine, all of which are normally suppressed in LC-FAODs (Figure 5D). Tissue free carnitine and C_8_-carnitine levels were also significantly increased (Figure 5, E and F). Together these data show that oral C_8_-carnitine distributes to key organs affected in LC-FAODs, where its conversion to C_8_-CoAs produces energy but also boosts free carnitine levels, which can relieve mitochondrial stress in LC-FAODs by promoting acylcarnitine efflux (4). The therapeutic potential of C_8_-carnitine was evidenced by gavaging LCAD-KO mice with 0.5 mg/g C_8_-carnitine just prior to an acute exercise challenge, significantly improving performance while also limiting lactic acidosis (Figure 5, G and H).

### Oral C_8_-carnitine improves muscle symptoms in CPT2-deficient, but not CrAT-deficient, mice.

**Figure 6:**
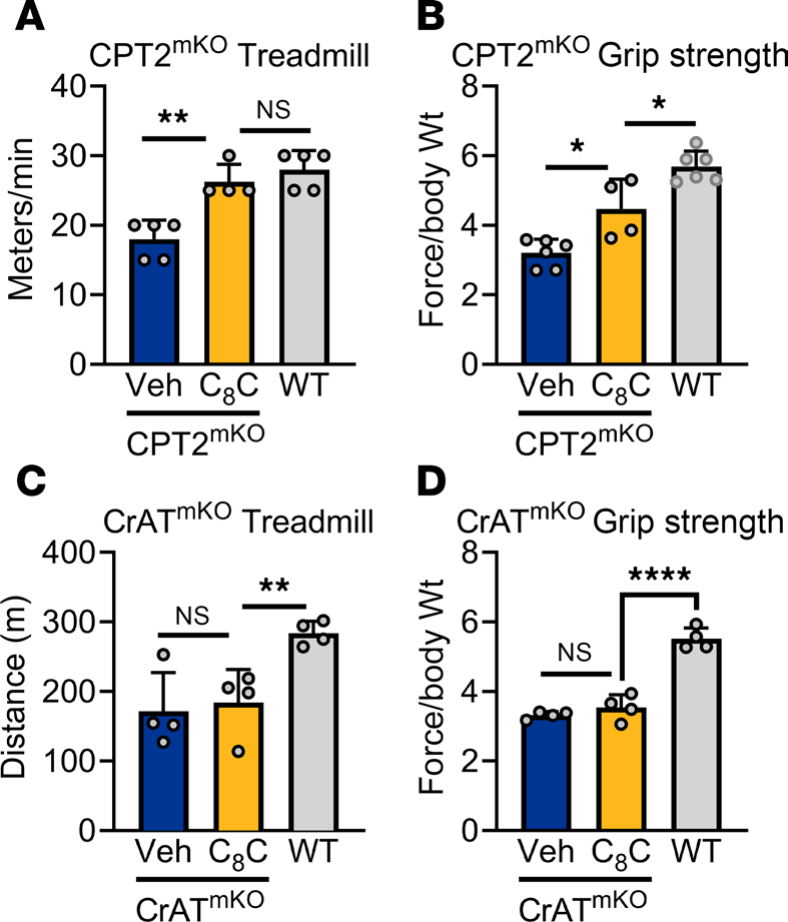
Oral C_8_-carnitine improves muscle symptoms in CPT2-deficient but not CrAT-deficient mice. (**A** and **B**) Muscle-specific KOs of CPT2 (CPT2^mKO^) were given saline vehicle (Veh) or C_8_-carnitine (C_8_C) at 0.5 mg/g 20 minutes before treadmill running (**A**) or grip strength testing (**B**). A group of WT littermates were assessed as control. A “top speed” running protocol was used, which assesses the highest velocity at which the mice could complete a 5-minute run. (**C** and **D**) The same experimental design as in **A** and **B**, but using CrAT muscle-specific knockouts (CrAT^mKO^). *P < 0.05; **P < 0.01; ****P < 0.0001 by Student’s *t* test, with Bonferroni correction for multiple comparisons. Data are shown as mean ± SEM.

Patients deficient in CPT2 present with many of the same symptoms as patients deficient in VLCAD, including exercise-induced decompensation and rhabdomyolysis. Our data presented in Figure 4 indicate that CPT2 is not required for muscle C_8_-carnitine oxidation, and therefore, C_8_-carnitine may have utility for treating CPT2 deficiency. Complete ablation of CPT2 in mice is embryonic lethal. Muscle-specific KOs exhibit much milder disease, characterized by compromised exercise performance (31). Using the single-dose treatment paradigm studied above in LCAD-KO mice, we observed that C_8_-carnitine significantly increases running speed and grip strength in CPT2^mKO^ mice (Figure 6, A and B). In contrast, muscle-specific CrAT^mKO^ mice did not show any improvement in these parameters after C_8_-carnitine dosing (Figure 6, C and D). This strongly suggests that CrAT, and not CPT2, is required for the therapeutic benefit of medium-chain acylcarnitines for treating LC-FAODs.

## Discussion

Our data reposition acylcarnitines from their well-characterized role as mitochondrial transport intermediates and diagnostic biomarkers into potential metabolic therapeutics. Specifically, we show that orally administered medium-chain acylcarnitines are absorbed into circulation intact, reach peripheral tissues, and are rapidly oxidized to provide energy. As an energy source, oral acylcarnitines have several advantages over MCT or triheptanoin. First, medium-chain acylcarnitines are water soluble and avoid the gastrointestinal complications associated with the consumption of oils. Second, acylcarnitine therapy not only supports oxidative metabolism but also releases free carnitine upon metabolism, helping to preserve the free CoA pool and thereby relieve mitochondrial stress. Third, whereas activation of free fatty acids such as C_7_ or C_8_ to their corresponding acyl-CoAs by ACSMs requires an input of ATP, conversion of acylcarnitines to acyl-CoAs by CrAT requires no energy. Together, these features offer a unique mechanism of action distinct from current therapies like triheptanoin.

For many years, MCT and triheptanoin have been promoted as alternative energy sources for LC-FAODs due to their ability to bypass the carnitine shuttle and rapidly fuel peripheral tissues. However, there is minimal empirical evidence to support their efficacy for treating muscle symptoms. Just one study examined the effect of MCT in a LC-FAOD mouse model (32). Using VLCAD-KO mice, this study showed that MCT mitigates postexercise long-chain acylcarnitine accumulation in skeletal muscle, consistent with improved metabolic tolerance during exertion. However, only biochemical parameters were measured, and therefore, any functional benefit regarding exercise performance was unclear. Similarly, in patients with LC-FAOD, MCT lowers heart rate at a fixed workload, raises circulating ketones, and improves metabolic control during exercise (33, 34). Again, while these changes are compatible with better exercise tolerance, they were not accompanied by statistically significant improvements in endurance metrics. For triheptanoin, a recent clinical trial reported improvements in 12-minute walk distance and cycle-ergometry output, but a lack of statistical significance makes interpretation of these data difficult (35). In pig hearts, direct infusion of C_7_ did not improve contractile power, suggesting that any cardiac benefit of C_7_ is secondary (36). We speculate that the highly variable effects of MCT and triheptanoin on exercise parameters observed in some patients is due to hepatic ketogenesis rather than direct oxidation of the administered fatty acids by working heart or muscle. This is supported by the work of the Brunengraber group, who used tracer methodologies to show that orally delivered triheptanoin does not reach circulation but is instead efficiently extracted by liver and oxidized to yield ketone bodies (17, 18, 37).

A major mechanistic insight from our study is the identification of CrAT, rather than CPT2, as the critical enzyme mediating the oxidation of medium-chain acylcarnitines in heart and skeletal muscle. This challenges existing assumptions about the uniformity of carnitine shuttle function across acyl-chain lengths. While both CPT2 and CrAT can catalyze the conversion of medium-chain acylcarnitines to medium-chain acyl-CoAs in vitro, only CrAT appears to do so in intact mitochondria, as evidenced by the complete loss of C_8_-carnitine oxidation in CrAT-deficient muscle but no change in CPT2-deficient muscle. CPT2 has 2 small membrane-binding helices that are thought to mediate interaction with cardiolipin and CACT, the acylcarnitine translocase that is imbedded within the inner mitochondrial membrane (38). Intriguingly, the CrAT 3-dimensional structure has an α helix (helix 1) that aligns spatially with the membrane-binding helices of CPT2 (38). The physiochemical properties of this helix indicate it has a highly hydrophobic surface that would be predicted to bind membranes (Supplemental Figure 5). We speculate that CrAT may bind to the inner mitochondrial membrane and form a functional complex with CACT to facilitate the import/export of medium-chain acylcarnitines, whereas CPT2 fulfills this role for long-chain substrates.

Historically, forward CrAT activity is designated as acyl-CoA + carnitine → acylcarnitine + CoA and reverse activity as acylcarnitine + CoA → acyl-CoA + carnitine. CrAT is active against substrates from 2 to 8 carbons in length (28, 29, 39, 40). Of these, only acetylcarnitine has been explored as a therapy. Acetylcarnitine went through multiple inconclusive clinical trials as a putative therapeutic for neurological diseases (41–43). The primary goal was to drive acetylcholine production. We postulate that manipulating the acetyl-CoA pool with exogenous acetylcarnitine is difficult due to a very large endogenous pool of acetylcarnitine (acetylcarnitine is > 90% of the body’s acylcarnitines). Additionally, acetyl-CoA is the metabolic currency of the cell, and its metabolism is very tightly regulated. Our innovation here was to use the upper end of CrAT’s substrate range, C_8_-carnitine, for which the endogenous pool is very small and for which a minimally regulated metabolic “sink” (medium-chain mitochondrial FAO) is in place to rapidly catabolize the acyl-CoA product and keep the CrAT reaction moving in the desired reverse direction. The concept of an active FAO sink may explain why cardiac mitochondria exhibited a clear preference for C_8_-carnitine over C_7_-carnitine while muscle mitochondria did not (Figure 3, A and B). The first FAO reaction for catabolizing the acyl-CoAs formed by CrAT is catalyzed by the acyl-CoA dehydrogenases. Between C_7_-CoA and C_8_-CoA, MCAD prefers the longer C_8_ substrate (44, 45), while C_7_-CoA is a substrate for both MCAD and short-chain acyl-CoA dehydrogenase (SCAD) (46). In our recently published multitissue proteomics dataset (47), the ratio of MCAD/CrAT is ~10 in heart versus 5 in muscle, while SCAD is much less abundant with a SCAD/CrAT ratio of ~1:1 in both heart and muscle. We postulate that the abundance of MCAD in heart, combined with its strong preference for C_8_-CoA as a substrate, may drive respiration on C_8_-carnitine preferentially over C_7_-carnitine. The role of downstream FAO enzymes in regulating flux through CrAT will be mechanistically interrogated in future studies.

Beyond LC-FAODs, medium-chain acylcarnitines may provide therapeutic value in a range of other metabolic disorders that have failed to respond to triheptanoin, such as McArdle’s disease, phosphofructokinase deficiency, GLUT1 deficiency, pyruvate carboxylase deficiency, and alternating hemiplegia of childhood (48–52). Unlike MCT or triheptanoin, oral acylcarnitines require no lipases for processing and, thus, may be absorbed from the gut very quickly, allowing for energy supplementation in disorders where pancreatic insufficiency is common, such as cystic fibrosis. Our findings also carry implications for the broader field of sports nutrition. Despite the widespread use of MCT oil for weight loss and athletic performance, our data reveal that muscle and heart mitochondria are ill equipped to directly metabolize medium-chain fatty acids due to a lack of ACSM expression. Most orally ingested MCT is sequestered by the liver, with little reaching peripheral tissues in usable form. In contrast, medium-chain acylcarnitines show greater systemic distribution and are efficiently utilized by muscle and heart. Importantly, long-term MCT use has been shown to increase liver triglyceride content (53), a side effect unlikely to be shared by acylcarnitine formulations, which cannot be directly elongated or stored like medium-chain free fatty acids. Therefore, medium-chain acylcarnitines may offer a more targeted, effective, and safer supplement for exercise performance and recovery.

In summary, our study redefines the therapeutic landscape of fatty acid oxidation disorders by unveiling a previously unappreciated metabolic bottleneck — the lack of ACSM expression in target tissues — and by introducing medium-chain acylcarnitines as a compelling alternative to MCT-based therapies. We uncover CrAT as the critical mediator of medium-chain acylcarnitine metabolism in muscle and heart and provide preclinical evidence that oral C_8_-carnitine is bioavailable, well tolerated, and effective. Future investigations will explore pharmacokinetics, long-term safety, and dosing strategies of medium-chain acylcarnitines. Structural studies to characterize CrAT-CACT interactions and tissue-specific delivery mechanisms will also be critical. Ultimately, this work opens therapeutic avenues not only for LC-FAODs but also for a wide range of metabolic and neuromuscular disorders where energy failure is central to disease pathogenesis.

## Methods

### Sex as a biological variable.

Our study examined male mice because male animals exhibited less variability in phenotype. It is unknown whether the findings are relevant for female mice.

### Animals.

Mice and rats studied through this work were maintained in a pathogen-free barrier facility on a 12-hour light/dark cycle. LCAD-KO mice were bred and genotyped as previously described (14). CPT2 and CrAT-cKO strains were provided by Michael Wolfgang (Johns Hopkins University, Baltimore, Maryland, USA) and Krisztian Stadler (Pennington Biomedical Research Center, Baton Rouge, Louisiana, USA) respectively, and have been well described (27, 54–57). To generate muscle-specific KO strains, both were crossed with the Cg-Tg(ACTA1-cre)79Jme/L transgenic strain (Jackson Laboratories, Bar Harbor, ME). All genotyping was performed by Transnetyx, Inc. (Memphis, TN).

### Acute and chronic lipid administration.

Pelleted rodent diets containing either MCT (C_8_ triglyceride) or triheptanoin (C_7_ triglyceride) were manufactured by Research Diets Inc. and were formulated to contain 35% of calories as MCT or triheptanoin, with an additional 10% of calories as long-chain fat to supply essential fatty acids. These diets were fed ad libitum for 10 days. For C_8_-carnitine feeding, C_8_-carnitine (ClearSynth) was mixed into powdered rodent diet at 3% w/w and fed in jars for 10 days. For acute administration of MCT, triheptanoin, and C_8_-carnitine, the lipids were either gavaged as the oils (MCT, triheptanoin) or dissolved in saline (C_8_-carnitine). For the latter, saline vehicle was given to the control animals. All dosing was 0.5 mg/g body weight, delivered 20 minutes prior to functional testing.

### Treadmill running.

All treadmill experiments used an Ugo Basile treadmill. Mice were acclimated to the machine by walking at 5 m/min for 20 minutes for 3 consecutive days prior to exhaustion testing. On the day of testing, mice were fasted for 5 hours beginning at 7 a.m. Blood lactate and glucose were tested with handheld meters just prior to running. The running protocol was optimized for each strain. For LCAD-KO, the treadmill was programmed to accelerate from a starting pace of 5 m/min to 25 m/min over a 10 minute period; after reaching the maximum speed of 25 m/min they were run to exhaustion, defined as taking 25 cumulative foot shocks over the course of the exercise or remaining on the shock grid for > 5 seconds. Muscle-specific CPT2 and CrAT knockout mice had much milder exercise intolerance than LCAD-KO mice. For these strains, a “top speed” running protocol was adapted from Petreyra et al. (31). After an initial walking pace at 5 m/min for 5 minutes, the mice were rested for 2 minutes; then, the speed was increased by increments of 5 m/min, with 2 minutes of rest in between each increment, until the mice reached a speed where they were unable to run for the full 5-minute trial, defined by the mouse taking 25 total foot shocks or remaining on the shock grid for more than 5 consecutive seconds. The “top speed” for each mouse was defined as the speed of the last successfully completed 5 minutes interval.

### Grip strength assessment.

Mice were fasted for 5 hours prior to testing. Baseline measurements were taken using a BIOSEB GT3 grip strength test machine (BIOSEB, North Pinellas, FL) fitted with a metal gripping grid, measuring grams of pull. Each mouse was held by the tail while the front legs were allowed to grip the grid. The tail was then gently pulled until the mouse released its grip. Each mouse was measured 5 times to gather a representative number of measurements. The pull data were normalized to body weight.

### Open field actimeter measurements.

The BIOSEB Infrared Actimeter system (BIOSEB, North Pinellas, FL) utilizes an X-Y-Z axis infrared laser system to track maximum speed, mouse locomotion, total distance traveled in actimeter, and the percent of time resting in the actimeter. Mice were put into the open field actimeter system after a 5-hour fast and locomotion metrics were recorded by the BIOSEB system for 20 minutes.

### ACSM expression.

First, we queried our previously published proteomics dataset (20) for ACSM proteins in WT, fed-state liver, quadriceps, and heart. The peak intensities for ACSM isoforms 1-5, from *n* = 5 male 129S1 mice, were plotted as a heatmap in Graphpad Prism 10.0. This data was verified by immunoblotting. In total, 50 μg of total liver, muscle, or heart proteins were electrophoresed on Criterion SDS polyacrylamide gels (BioRad, Hercules, CA) and transferred to nitrocellulose membranes. Primary antibodies used were anti-GAPDH (Proteintech, 10494-1-AP, 1:1000 dilution), anti-ACSM1 (Invitrogen, PA5-32119, 1:1000), anti-ACSM2 (Abcam, ab181865, 1:500), anti-ACSM3 (Invitrogen, PA5-100374, 1:1000), and anti-ACSM5 (Proteintech, 16591-1-AP, 1:1000). Membranes were incubated overnight at 4°C with primary antibodies, then incubated with HRP-conjugated secondary Goat Anti-Rabbit or Goat Anti-Mouse IgG antibody (Bio-Rad, 1706515 and OBT1500P, Hercules, CA) at 1:2000 dilution for 1-2 hours at room temperature. Clarity Max ECL kit (Bio-Rad, Hercules, CA) was used to visualize protein bands and images of the membranes were captured using the Protein Dock system (FluorChem M System, Bio-Techne, Minneapolis, MN).

### Tissue acylcarnitine analysis.

LCAD-KO mice were fed either powdered standard rodent diet or the same diet mixed with C_8_-carnitine (3% w/w) for 10 days. All mice were sacrificed in the fed state, early in the light cycle. Liver, heart, and quadriceps tissue were snap frozen for acylcarnitine profiling in the Metabolic Core Facility at the University of Pittsburgh/UPMC Children’s Hospital of Pittsburgh as described (58, 59). Frozen tissue (30–60 mg) was wrapped in aluminum foil and suspended in a liquid nitrogen bath for 2 minutes, then removed, placed on a hard benchtop and the tissue pulverized with a hammer. The pulverized tissue was transferred to a preweighed microcentrifuge tube and a postweight determined. An aliquot (20 μL) of isotope labeled carnitine standards (reconstituted in methanol) was spiked into the tube along with 280 μL of ethanol (abs.), then vortex-mixed for 1 minute to extract the acylcarnitines. The tube was placed in a sonication ice water bath (4 – 22°C) and sonicated for 30 minutes with vortex-mixing (30 sec every 10 min). After sonication, 700 μL of ethanol (abs.) was added to the tube and vortex-mixed for 30 sec. The extracted sample was centrifuged (13,000xg, 10 min, 4°C). A portion of the supernatant (50 μL) was dried under a stream of nitrogen gas and the acylcarnitine butyl esters generated by reaction (60°C for 15 min) in 100 μL of 3 N HCl in butanol. Dried residues were reconstituted in acetonitrile/water (80:20) for flow-injection ESI-MS/MS analysis. Analysis was performed on a triple quadrupole API4000 mass spectrometer (AB Sciex™, Framingham, MA) equipped with an ExionLC 100 HPLC system (Shimadzu Scientific Instruments, Columbia, MD). Acylcarnitine standards were purchased from Amsterdam UMC—VUmc (Amsterdam, NL) and Cambridge Isotope Laboratories, Inc. (Andover, MA). Acylcarnitines were measured using multiple reaction monitoring (MRM) for free carnitine (C_0_, m/z 218 → m/z 103) and acetylcarnitine (C_2_, m/z 260 → m/z 85) and precursor scan for precursor ions (Q1) of acylcarnitines (C_3_ to C_18_, scan range m/z 270 to 502) that generated a product ion (Q3) at m/z 85.

### Mitochondrial isolation.

For Oroboros respirometry, fresh mitochondria were isolated from mouse skeletal muscle, heart, and liver using differential centrifugation. All protocols for mitochondrial isolation were adapted from MitoPedia, the official Oroboros reference site (https://wiki.oroboros.at/index.php). After tissues were excised from the mice, all steps were carried out on ice or in 4°C conditions. Conditions varied slightly for each tissue type as follows. Skeletal muscle: tissue was placed in ice cold phosphate buffered saline (PBS) containing 10 mM EDTA, minced and incubated in PBS with 10 mM EDTA and 0.05% Trypsin for 30 minutes on ice to permeabilize the tissue. Tissue was centrifuged at 200*g* to remove the trypsin buffer and replaced with IBM1 buffer containing 50 mM KCl, 67 mM sucrose, 50 mM Tris pH7.4, 10 mM EDTA, and 0.2% BSA before homogenization via mechanical shearing using a glass Dounce homogenizer. The homogenate mix was centrifuged at 700*g* for 10 minutes to remove cellular and tissue debris. Then, the mitochondria-containing supernatant was transferred to a new tube and centrifuged at 8,000*g* for 10 minutes. The mitochondrial pellet was resuspended in IBM2 buffer containing 250 mM sucrose, 3 mM EGTA and 10 mM Tris pH 7.4 before a final 8,000*g* centrifugation for 10 minutes. Resulting intact mitochondria were resuspended in Elution Buffer containing 250 mM sucrose, 0.02 mM EGTA, 10 mM Tris pH 7.4, 2 mM Tris-phosphate pH 7.4, and 5 mM MgCl_2_. Heart: freshly isolated hearts were washed 3 times with ice cold PBS to remove excess blood before being placed in Buffer A containing 180 mM KCl, 4 mM EDTA, and 10 mg of BSA. Heart tissue was then minced and rinsed with ice cold Buffer B containing 180 mM KCl, 4 mM EDTA, 10 mg of BSA, and 2.5 mg subtilisin to rinse off any remaining Buffer A before being incubated on ice in Buffer B for 10 minutes to allow the subtilisin to act as a detergent. After 5 minutes of centrifuging at 200*g*, minced heart tissue was resuspended in Buffer A and homogenized by mechanical shearing. Cell and tissue debris were pelleted by 1000*g* centrifugation for 10 minutes, after which the supernatant was transferred to a fresh tube and centrifuged at 6,200*g* for 10 minutes. Intact mitochondria were resuspended in Buffer C containing 180 mM KCl, and 4 mM EDTA. In the liver, fresh tissue was excised into ice-cold buffer containing 225 mM mannitol, 75 mM sucrose, and 0.2 mM EDTA. The liver was then minced and homogenized by mechanical shearing before a 10-minute centrifugation at 1,000*g*. The supernatant was transferred to a fresh tube and centrifuged for 10 minutes at 6,200*g*. Intact mitochondria were resuspended in Buffer C, described above, centrifuged once more at 6,200*g* for 10 minutes to wash the mitochondria, and finally resuspended in Buffer C, described above.

### Mitochondrial respirometry.

An Oroboros Oxygraph-2K was used to assess the level of respiration by freshly isolated mitochondria (described above). In total, 30 μL of liver, heart or skeletal muscle mitochondria was added to O_2_-equilibrated chambers containing 2 mL of MiR05 buffer (0.5 mM EGTA, 3 mM MgCl_2_, 60 mM lactobionic acid, 20 mM taurine, 10 mM potassium diphosphate, 20 mM HEPES, 110 mM D-sucrose). Once the oxygen concentration and change in oxygen concentration became stable at baseline, 10 μM of cytochrome C (Sigma, St. Louis, MO) was added to both chambers to assess mitochondrial membrane integrity. Subsequently, 5 mM malate (Sigma, St. Louis, MO) was added to both chambers to support oxaloacetate production, fueling the TCA cycle. In total, 2 mM ADP was then added to support ATP production from the electron transport chain. Finally, either 50 μM of medium-chain free acids or acylcarnitines were added and maximal respiration response recorded. An *n* = 4 or more was performed for all comparisons. All Oxygraph-2K signals were normalized to mitochondrial protein content as determined by BCA protein assay kit (Thermo Fisher Scientific, Waltham, MA).

### Lipid oral bioavailability.

Sprague Dawley Rats fitted with externalized jugular access ports were purchased from Charles River (Wilmington, MA). The experimental design was to dose with 0.5 mg/g of either triheptanoin or C_8_-carnitine, comparing blood concentrations over time following i.v. doses to those of oral doses. For oral dosing, rats were briefly incapacitated using isoflurane and then gavaged with 0.5 mg/g of body weight of either triheptanoin or C_8_-carnitine via oral gavage. The rats quickly regained consciousness. Blood samples were drawn from the jugular i.v. port of each awake, dosed rat at t=0 minutes, 10 minutes, 20 minutes, 30 minutes, 60 minutes, 90 minutes, and 120 minutes. For i.v. dosing, triheptanoin oil was not used as i.v. administration of oils is toxic. Rather, an equal amount of C_7_ free fatty acid was dissolved in 0.9% saline solution and injected into the port of awake rats. C_8_-carnitine was administered in the same manner. All blood samples were centrifuged at 1,500xg for 10 minutes, and serum was snap-frozen and stored at –80°C until time of analysis by mass spectrometry. Serum samples were analyzed via tandem mass spectrometry (MS/MS) for free fatty acid quantification by the Mass Spectrometry Core at the University of Pittsburgh, or for serum acylcarnitine profiling by the Metabolic Core at the University of Pittsburgh/UPMC Children’s Hospital of Pittsburgh. Prior to C_7_ analysis, the serum lipids were hydrolyzed to release any C_7_ from intact triglycerides.

### In vivo ^14^C-C_8_-carnitine oxidation to ^14^C-CO_2_.

WT male mice were fasted for 5 hours, then administered a bolus of C8-carnitine dissolved in saline (0.5 mg/kg) that also contained radiolabeled ^14^C-C_8_-carnitine as a tracer (3 μCi/mouse). Individual mice were placed into a sealed box with an air intake port at one end and connected to a vacuum from the other end. The vacuum pulled exhaled air out of the box, and fresh air simultaneously into the box. The exhaled air was passed through a flask containing 1M KOH to trap CO_2_. Every 10 minutes a sample of KOH was withdrawn from the flask and subjected to scintillation counting to quantify the accumulating ^14^C-CO_2_.

### ACSM activity.

Initial assays with C_8_ followed the conversion of ^14^C-C_8_ into ^14^C-C_8_-CoA as we previously described (53). Briefly, mitochondria were isolated by differential centrifugation and resuspended in cold SET buffer (10 mM Tris-HCl, 250 mM sucrose, 1 mM EDTA). Synthetase reactions (200 μL) contained 5 μL of homogenate with 10 μM ^14^C-fatty acid in a buffer of 40 mM Tris–HCl, 5 mM ATP, 5 mM MgCl_2_, 4 mM CoA, 0.8 mg/mL Triton WR1339, and 1 unit/mL inorganic pyrophosphatase. After 2-minute incubation at 37°C, reactions were stopped with sulfuric acid and extracted 4 times with ether prior to scintillation counting. Data were normalized to protein concentration. Later experiments used tandem mass spectrometry to directly detect the acyl-CoAs formed in the reactions. Freshly prepared tissue homogenates (250 μg) were incubated with 50 μM of either free fatty acid (C_7_, C_8_, C_16_) or the acylcarnitine conjugates of these fatty acids, in a reaction buffer containing 40 mM Tris (pH 8), 5 mM ATP, 5 mM MgCl_2_, and 1 U/mL inorganic pyrophosphatase. The reactions were started by addition of free CoA to a final concentration of 200 μM. Reaction tubes were placed in a water bath shaker at 37°C for one hour. Reactions were then flash frozen in liquid nitrogen and stored at –80°C. The medium-chain CoA concentration of each reaction tube was measured via tandem MS/MS analysis at the Biochemical Genetics Laboratory at Colorado’s Children’s Hospital (Aurora, Colorado, USA).

### Carnitine acyltransferase expression, purification, and activity.

Expression constructs for human CrAT (pJH15-pET21-CRAT-6His; a gift from Chaitan Khosla, Stanford University, Palo Alto, California, USA) and human CPT2 (pET21b-CPT2-6His) were transformed into *E*. *coli* and expressed overnight at 18°C, by addition of 0.5 mM IPTG. Bacterial pellets were lysed via sonication and the lysates clarified by ultracentrifugation at 40,000xg for 60 minutes. Supernatants were applied to a preequilibrated ion-exchange chromatographic column HisTrap HP on an ӒKTA pure chromatography system (Cytiva). The column was washed 20 column volumes of washing buffer (50 mM phosphate, 10 mM imidazole, 300 mM NaCl, 10% glycerol, pH 8). Recombinant protein was eluted by increasing the percentage of elution buffer (50 mM phosphate, 250 mM imidazole, 300 mM NaCl, 10% glycerol, pH 8). Eluted pure CrAT and CPT2 proteins were dialyzed against above phosphate buffer without any imidazole and then concentrated and kept frozen in small aliquots at –80°C. Enzyme activity in the direction of acylcarnitine → acyl-CoA was measured with a coupled assay using the fluorescence of electron transferring flavoprotein (ETF) as the readout. In this assay, illustrated in Supplemental Figure 6, recombinant CrAT or CPT2 were incubated with C_7_-carnitine or C_8_-carnitine to generate C_7_-CoA or C_8_-CoA, which were then dehydrogenated by recombinant MCAD, which passes electrons to its natural electron acceptor ETF. ETF is a fluorescent protein which becomes quenched upon accepting electrons. We have previously published a method using recombinant ETF to follow the activity of acyl-CoA dehydrogenases (60). Here, each 200 μL reaction contained 50 mM Tris (pH 8), 0.5% glucose, glucose oxidase (~20 U/mL), catalase (~10 U/mL), 2 μM recombinant ETF, 1 μg recombinant MCAD, 0.2 μg recombinant CPT2 or CrAT (MCAD/CPT2 or MCAD/CrAT ratios were 5:1), and 0.5 mM CoA. After incubation at 32° for 2 minutes, baseline fluorescence was recorded for 1 minute at Ex 340 nm/Em 490 nm on a FLUOstar Omega plate reader (BMG Labtech). Then, the reaction was started by addition of C_7_-carnitine or C_8_-carnitine to a final concentration 25 μM and the fluorescence intensity was recorded for 1 minute. The slope and *y* intercept were used to calculate activity as we have previously described (60).

### Statistics.

All statistical analyses were performed in GraphPad Prism (version 10.3, Graphpad Prism Software Inc.). Two-tailed Student’s *t* tests were used to compare treated to untreated, KO to WT, and other 2-group comparisons. An AUC calculation was used to determine the oral and i.v. bioavailability of triheptanoin and C_8_-carnitine. After constructing a ratio of oral AUC/i.v. AUC, a *t* test was used to compare the calculated overall bioavailability. Oroboros oxygraph data were generated in DatLab (version 8.0, Oroboros Instruments) and exported to Microsoft Excel where the average peak difference in oxygen consumption was calculated. All data were subsequently transferred to Prism for Student’s *t* test analysis.

### Study approval.

All protocols for animal use and experiments were approved by the University of Pittsburgh IACUC, ensuring all work was done in accordance with the Animal Welfare Act and Public Health Service Policy on Humane Care and Use of Laboratory Animals.

### Data availability.

All data are included in the Supporting Data Values file.

## Author contributions

ESG and KJS conceptualized the studies. The manuscript was written by ESG and KJS and then edited by YZ, CVL, and TCW. KJS bred and maintained the mouse colonies and performed all of the animal experiments, assisted by BBZ, ACR, and AVS. SSB performed the radioactivity-based assay for C8-CoA synthesis and assisted KJS with Oroboros respirometry. CVL analyzed tissue and serum acylcarnitines by mass spectrometry, while ODA and TCW quantified acyl-CoAs by mass spectrometry. ACR and YZ expressed and purified recombinant CPT2 and CrAT, and YZ performed enzyme activity assays on these recombinant enzymes.

## Funding support

This work is the result of NIH funding, in whole or in part, and is subject to the NIH Public Access Policy. Through acceptance of this federal funding, the NIH has been given a right to make the work publicly available in PubMed Central.

- NIH grants DK090242 and HD103602 (ESG).
- The Pittsburgh Liver Research Center (DK120531).
- University of Pittsburgh, Department of Pediatrics.

## Supplementary Material

Supplemental data

Unedited blot and gel images

Supporting data values

## Version 1 10/16/2025

In-Press Preview

## Version 2 11/24/2025

Electronic publication
