# Supplementary figures and images for: Oral octanoylcarnitine alleviates exercise intolerance in mouse models of long-chain fatty acid oxidation disorders
Source: JCI Insight. 2025 Oct 16;10(22):e199443. doi: 10.1172/jci.insight.199443 (PMC12643506; doi:10.1172/jci.insight.199443)

Unedited blot images for Figure 4D

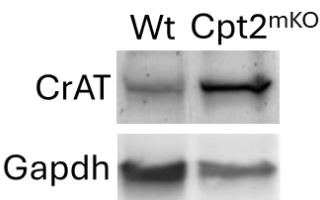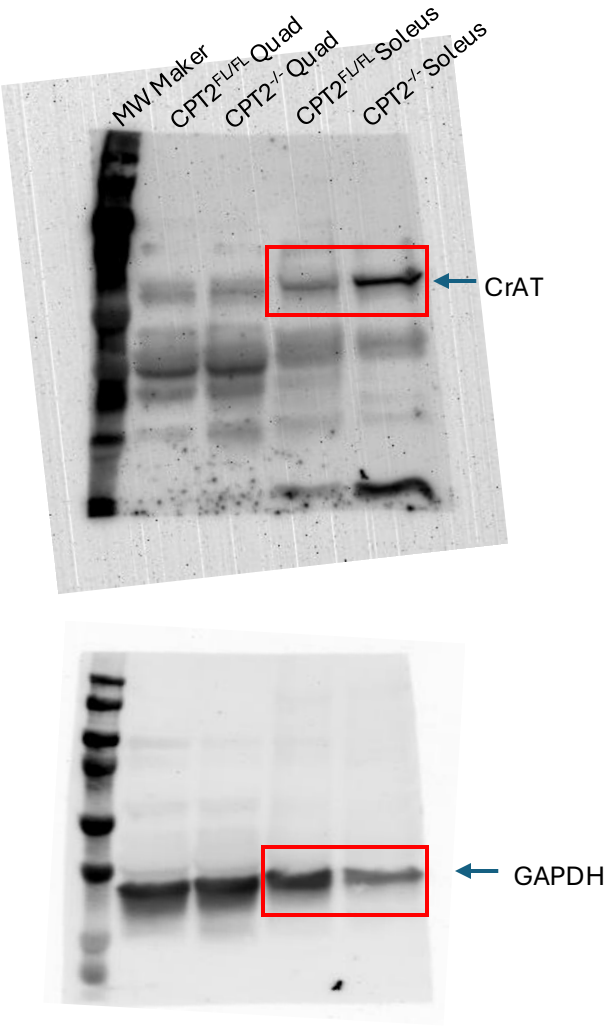

Supplement: Unedited blot and gel images [file jciinsight-10-199443-s212.pdf]
